# Frontiers of conservation

**DOI:** 10.1111/cobi.14432

**Published:** 2024-12-22

**Authors:** Yves Meinard, Jean‐Yves Georges

**Affiliations:** ^1^ Aix‐Marseilles Université, CNRS Centre Gilles Gaston Granger (UMR 7304) Aix‐en‐Provence France; ^2^ Université de Strasbourg, CNRS, IPHC (UMR 7178) Strasbourg France

**Keywords:** anthropogenic impacts, conservation, culture, decision‐making, nature, protected areas, áreas protegidas, conservación, cultura, impactos antropogénicos, toma de decisiones, naturaleza

## Abstract

Action‐oriented conservation sciences are crippled by 3 false assumptions. First, although it is recognized in theory that natural and anthropic components of ecosystems are tightly intertwined, in practice, many conservation policies and actions are still based on the assumption that human and nonhuman stakes should be dealt with in deeply different ways. Second, although the anchorage of environmental sciences in values is amply demonstrated, many conservation scientists still assume they will lose their scientific credentials if they actively participate in decision‐making. Finally, although there is much scientific evidence of the permeability—to both protected entities and threats—of static geographic frontiers delimiting protected areas, many conservation policies are still based on the assumption that these frontiers in themselves produce relevant protections. To overcome these false assumptions, it is useful to articulate them in terms of *frontiers* based on 2 ideas associated with the term. As a synonym of *border*, *frontier* materializes a limit whose crossing can have high stakes. As used in phrases such as *frontiers of knowledge*, the term also refers to the ever‐moving horizon of what should be overcome. These 2 ideas capture the reasons current attempts at overcoming the 3 assumptions remain unsatisfactory. They are also useful for elaborating a new vision of conservation to simultaneously break from the 3 assumptions. Instead of taking fixed geographic frontiers of protected areas for granted, conservation scientists should participate, along with stakeholders and Indigenous peoples, in the collective identification of the conservation problems that need to be addressed. For these problems, decision committees that include representatives of concerned humans and representatives of concerned nonhumans should be formed to determine the temporal and spatial scope of relevant conservation actions. The result would be multidimensional protected areas dynamically fine‐tuned to the conservation issues they address and to changing environmental conditions.

## Abstract (es)

Fronteras de la conservación

**Resumen:**

Las ciencias de la conservación orientadas hacia la acción adolecen de tres suposiciones falsas. Primero, aunque en teoría se reconoce que los elementos naturales y antropogénicos de los ecosistemas tienen una fuerte conexión, en la práctica muchas de las políticas y acciones de conservación todavía se basan en el supuesto de que los intereses humanos y no humanos deberían abordarse de maneras muy diferentes. Segundo, aunque se ha demostrado ampliamente lo ancladas que están las ciencias ambientales en los valores, muchos científicos de la conservación todavía asumen que perderán sus credenciales científicas si participan activamente en la toma de decisiones. Por último, aunque existe mucha evidencia científica de la permeabilidad, tanto hacia las entidades protegidas como hacia las amenazas, de las fronteras geográficas estáticas que delimitan las áreas protegidas, muchas políticas de conservación todavía se basan en el supuesto de que estas fronteras producen una protección relevante por sí mismas. Para sobrepasar estas suposiciones es útil articularlas en términos de las fronteras basadas en dos ideas asociadas al término. Como sinónimo de margen, la frontera materializa un límite que, al cruzarse, puede representar un gran desafío. Si se usa en frases como fronteras del conocimiento, el término también se refiere al horizonte móvil de lo que a uno debe sobreponerse. Estas ideas representan las razones por las que todavía no son satisfactorios los intentos actuales de sobreponerse a las tres suposiciones falsas. Estas ideas también son útiles para construir una nueva visión de la conservación que se separa simultáneamente de las tres suposiciones. En lugar de dar por hechas las fronteras geográficas de las áreas protegidas, los científicos de la conservación deberían participar junto con los actores y pueblos indígenas en la identificación colectiva de los problemas de conservación que se necesitan abordar. Deberían formarse comités decisorios que incluyan representantes de los humanos y no humanos involucrados para determinar el enfoque espacio‐temporal de las acciones relevantes de conservación para los problemas mencionados. El resultado serían áreas protegidas multidimensionales con ajustes dinámicos a los temas de conservación que abordan y para las condiciones ambientales cambiantes.

## INTRODUCTION

Many conservation policies place marked emphasis on delineating protected areas as a key conservation tool. A prominent example of this tendency is the pledge by 116 countries to protect 30% of all land and water by 2030 (30×30 target) as part of COP15 on biodiversity, one of the most salient international agreements on biodiversity in recent decades. This goal refers to both protected areas explicitly pursuing biodiversity conservation objectives, such as national parks or nature reserves, and “other effective area‐based conservation measures” (OECMs) (Gurney et al., 2021) (i.e., managed areas that happen to sustain biodiversity even though this is not their original objective). Such a pledge seems to contrast with the usual inability of global decision makers to act decisively by considering scientists’ warnings about anthropogenic impacts on the environment (Ripple et al., 2017). However, it is unclear how the areas in question are supposed to be protected from some of the major drivers of biodiversity loss—climate change, widespread pollution, and biological invasions (Brahney et al., 2020; Cuff, 2023; Meng et al., 2023; Rowan & Rowan, 2023). Also unclear is how implementing such protections can avoid infringing on rights of Indigenous peoples and local communities. The real ambition behind the 30×30 political slogan, therefore, raises questions (Obura, 2023).

If pledges such as COP15's are to become reality, there is a need to rethink the way protected areas are defined and governed. This involves rethinking 3 false assumptions that currently cripple conservation sciences in practice. First, it is widely recognized in theory that natural and anthropic components of ecosystems are tightly intertwined. In practice, however, many conservation policies and actions are still based on the assumption that human and nonhuman stakes should be dealt with in deeply different ways. In that sense, crossing the natural–anthropic frontier remains a challenge.

Second, the anchorage of environmental sciences in values is amply demonstrated. However, many conservation scientists still assume they will lose their scientific credentials if they actively participate in decision‐making. Hence, many remain blinded by their belief that the knowledge–activism frontier is impermeable.

Third, there is much scientific evidence of the permeability—to both fluxes in protected entities and threats endangering them—of fixed geographic frontiers delimiting protected areas. However, many conservation policies are still based on the assumption that these frontiers in themselves unquestionably produce relevant protections (geographic frontier).

The term *frontiers* refers to intellectual obstacles in the first and second assumptions and to concrete material or administrative boundaries in the third assumption. We selected this term as the foundation of our argument because it carries with it ideas that can help overcome the 3 false assumptions in a synergistic way.

Two ideas associated with *frontier* are of particular relevance. First, as a synonym of *border*, a *frontier* materializes a limit whose crossing, by different entities in different directions and under different conditions, is high stakes. Referring to something as a frontier hence emphasizes the fact that thinking through and managing inward and outward crossings is important. Second, as used in phrases such as *frontiers of knowledge*, *frontiers* also refers to the ever‐moving horizon of what should be overcome. Referring to something as a frontier hence entails seeing it as something that is ever evolving and bound to remain a challenge.

These 2 ideas lie at the core of the 2 main meanings of *frontiers* in ordinary language, as explicated by, for example, *The Oxford English Dictionary* (oed.com) and *The American Heritage Dictionary* (ahdictionary.com). In various strands of the academic literature, including contributions focusing on conservation science and conservation issues, the term *frontiers* is sometimes given more specific meanings (e.g., Buchadas et al., 2022). We use the term in its ordinary, wide sense and as it is structured by the 2 ideas above.

Our claim is neither to have discovered how problematic the 3 false assumptions are nor to be the first to elaborate ideas to overcome them (forrunners and existing initiatives are duly referred to but not exhaustively treated). Rather, our claim is that there is added intellectual and practical value in thinking through these assumptions in terms of frontiers, i.e., as limits whose crossing in opposite directions is high stakes and as ever‐evolving challenging horizons.

Our use of *frontiers* is hence metaphorical, in the sense that we emphasize connotations associated with its different meanings. Although due caution is in order when using metaphorical meanings (Cachelin et al., 2010), they play important roles in conservation thinking. Metaphors can be used to mobilize decision makers and the public (Barua, 2011; Neilson, 2018). They have historically played important roles in structuring scientific explanations, in particular in biological sciences (Fox Keller, 2002). More generally, metaphors can be used as linguistic tools to identify hidden conceptual links or elaborate conceptual innovations (Ricoeur, 1977). This creative yet rigorous use of metaphorical meaning proves useful to strengthen existing conceptual and practical initiatives so that the 3 frontiers (i.e., natural–anthropic, knowledge–activism, and geographic frontiers) can be rethought.

## THE NATURAL–ANTHROPIC FRONTIER

Elaborating and implementing ways for human beings to protect nonhuman species and ecosystems from anthropogenic impacts appears to presuppose a clear distinction between what is natural (those affected by impacts) and what is anthropic (impacts and means to alleviate them).

**FIGURE 1:**
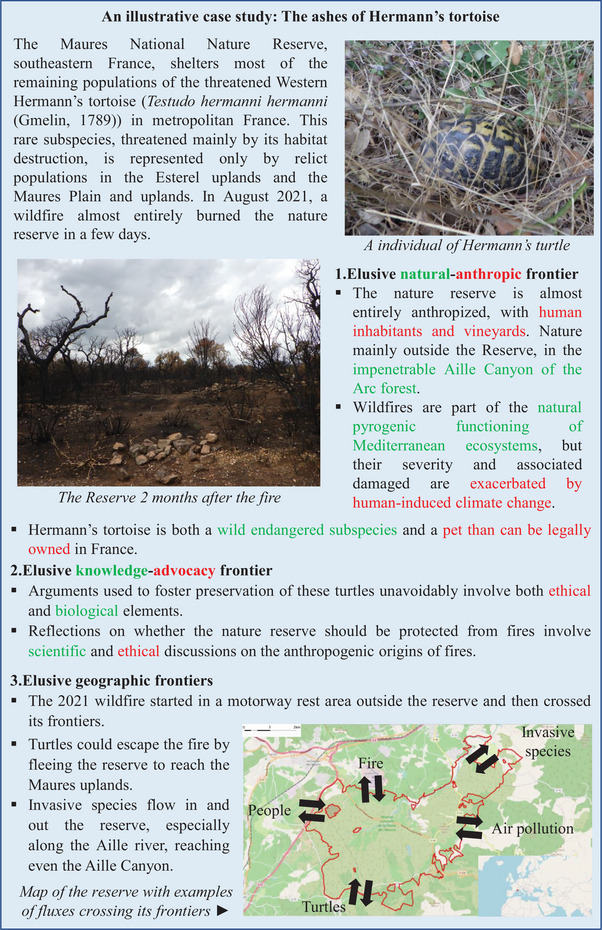
The 3 elusive frontiers of conservation illustrated by the case of Hermann's tortoise in the Maure National Nature Reserve (France).

However, as presented in an immense literature, innumerable examples from around the world concerning a variety of taxa and ecosystems illustrate the elusiveness of this assumption. Populations of Western Hermann's tortoise (*Testudo hermanni hermanni* [Gmelin, 1789]) (Zenboudji et al., 2016) decimated by a wildfire in a French nature reserve in 2021 illustrate this entanglement at 2 levels (Figure 1). These tortoises are both natural, by being a wild subspecies, and anthropic, by being legally kept as pets. The fire that decimated those populations is part of the natural pyrogenic functioning of Mediterranean ecosystems and was exacerbated by anthropogenic climate change. A similar natural–anthropic entanglement is illustrated by the classic case of patches of forests in Guinea. Long considered natural vestiges of the upper Guinean Forest, their presence is now known to be due to traditional human practice (Fairhead & Leach, 1996, 1998). Similarly, attacks by wolves during the Hundred Years’ War, long seen as an era‐defining human–nature conflict, are in fact behavioral consequences of human activities in a warfare context (Morizot, 2022). At a more general level, non‐native invasive species all entail a natural–anthropic entanglement: they existed before anthropogenic activities and globalization, but their current level of impact would not exist without the latter (Cuthbert et al., 2020).

Environmental sciences to some extent acknowledge the permeability of the natural–anthropic frontier (Ducarme & Couvet, 2020). Alternative worldviews inspired by Indigenous peoples highlighting this permeability are increasingly integrated in conservation thought and policy (Raymond et al., 2023). However, most action‐oriented attempts to rethink this frontier focus mainly on including partly anthropic entities as targets of conservation efforts, on a par with so‐called natural populations and species. This is the case with: arguments in favor of replacing wilderness with the purportedly more inclusive concept of wildness (Hinchliffe, 2008); arguments championing the valuation and preservation of “everyday biodiversity”; the “people and nature” paradigm (Mace, 2014); attempts under so‐called new conservation (e.g., Schilthuizen, 2018) to value and preserve the anthropogenic “new wild” (Pearce, 2015) or novel ecosystems (Hobbs et al., 2006, 2009); and “convivial conservation” (Büscher & Fletcher, 2019, 2020). A concrete example of this thinking is the conservation of meadows or pastures whose very existence in the absence of megaherbivores involves human interventions.

By including new entities among those that benefit from conservation actions, these conceptual and practical innovations are major contributions to conservation thought and practice. However, they cross the natural–anthropic frontier in only one direction (by including anthropic elements as conservation targets) and thereby arguably downplay the complementary need to cross it in the other direction (by including nonhuman entities as conservation decision makers). Besides, by championing the inclusion of ever‐more anthropic entities among those that benefit from conservation, these approaches tend to downplay the importance of respecting nature's independence (Maris, 2018) or otherness (Dereniowska & Meinard, 2021) vis‐à‐vis human beings. Indeed, one cannot acknowledge this independence or otherness as sources of conservation value if one sees natural anthropic as a divide that can and should be evacuated once and for all.

Despite undeniable advances, research agendas adressing the natural‐anthropic frontier hence remain unfinished. Progress can be made, first, by seeing crossings in both directions as high stake and, second, by seeing the divide as the ever‐evolving locus of reflections on what deserves to be conserved and why.

## THE KNOWLEDGE–ACTIVISM FRONTIER

Despite numerous debates on ethics in conservation (Baard, 2022; Biasetti et al., 2023; Latombe et al., 2022), most conservation scientists make a point of limiting their interventions in collective decision‐making to providing purportedly neutral information to decision makers through advisory panels (e.g., IPCC or IPBES at international scale, scientific councils of protected areas at more local scales) (Arpin et al., 2016). This self‐limitation of providing neutral information to decision makers is based on the assumption that the knowledge produced by conservation sciences can be value neutral. This assumption is untenable. Science and technology studies have highlighted the elusiveness of this value neutrality of scientific knowledge for decades (Granjou & Arpin, 2015; Saltelli et al., 2020). Practice teaches a similar lesson. When working on conservation projects, most people's ethical views evolve because they become aware of new aspects of the problems they tackle (Bain & Bongiorno, 2020; Bloom, 2010; Meinard & Cailloux, 2020). Hence, knowledge contributes to critical discussions on and elaborations of the values that can then be endorsed by activists, and activism contributes to knowledge by posing new questions and challenging values hidden in seemingly neutral knowledge.

Although they relentlessly emphasize the elusiveness of value neutrality, academics in environmental humanities are as timorous as their counterparts in biological sciences in their typical refusal to participate in decision‐making in environmental policies. This refusal is deleterious on 3 counts. First, because science is unavoidably anchored in values, by refusing to engage openly in decision‐making, conservation scientists end up indulging in a form of “stealth advocacy” (Cardou & Vellend, 2023). This consists, for them, in championing values while making it look as though they are purely value neutral. Such an attitude ultimately leaves science open to attack. Second, other actors—potentially with deleterious interests and goals—do not hesitate to participate in decision‐making. Hence, by refusing to participate, conservation scientists yield the ground to their adversaries. Third, because policy makers have by and large ignored scientists’ warnings for decades, the refusal to engage decisively in decision‐making has become increasingly untenable. It is hypocritical of scientists to deplore inaction when they are too hesitant to act themselves (Bassett, 2020).

Although avoiding participation in decision‐making is deleterious, a generalized involvement of scientists in decision‐making would run the risk of entrenching a technocracy. The need to acknowledge that knowledge and activism dynamically support each other should hence be accompanied by a critical awareness of possible drifts.

The use of *frontiers* hence appears relevant to characterize knowledge and activism because this term usefully captures the strengths and shortcomings of current discussions. Indeed, the practice of avoiding direct involvement in decision‐making reflects a limited awareness of how the frontier between knowledge and activism is crossed in both directions in most conservation projects. This echoes the first idea associated with *frontiers* (i.e., a limit whose crossing is high stakes). Similarly, the second idea, according to which frontiers are ever‐evolving and bound to remain a challenge, captures the need to develop a critical awareness of possible drifts in scientists’ attempts at participating in decision‐making.

## GEOGRAPHIC FRONTIERS

**FIGURE 2:**
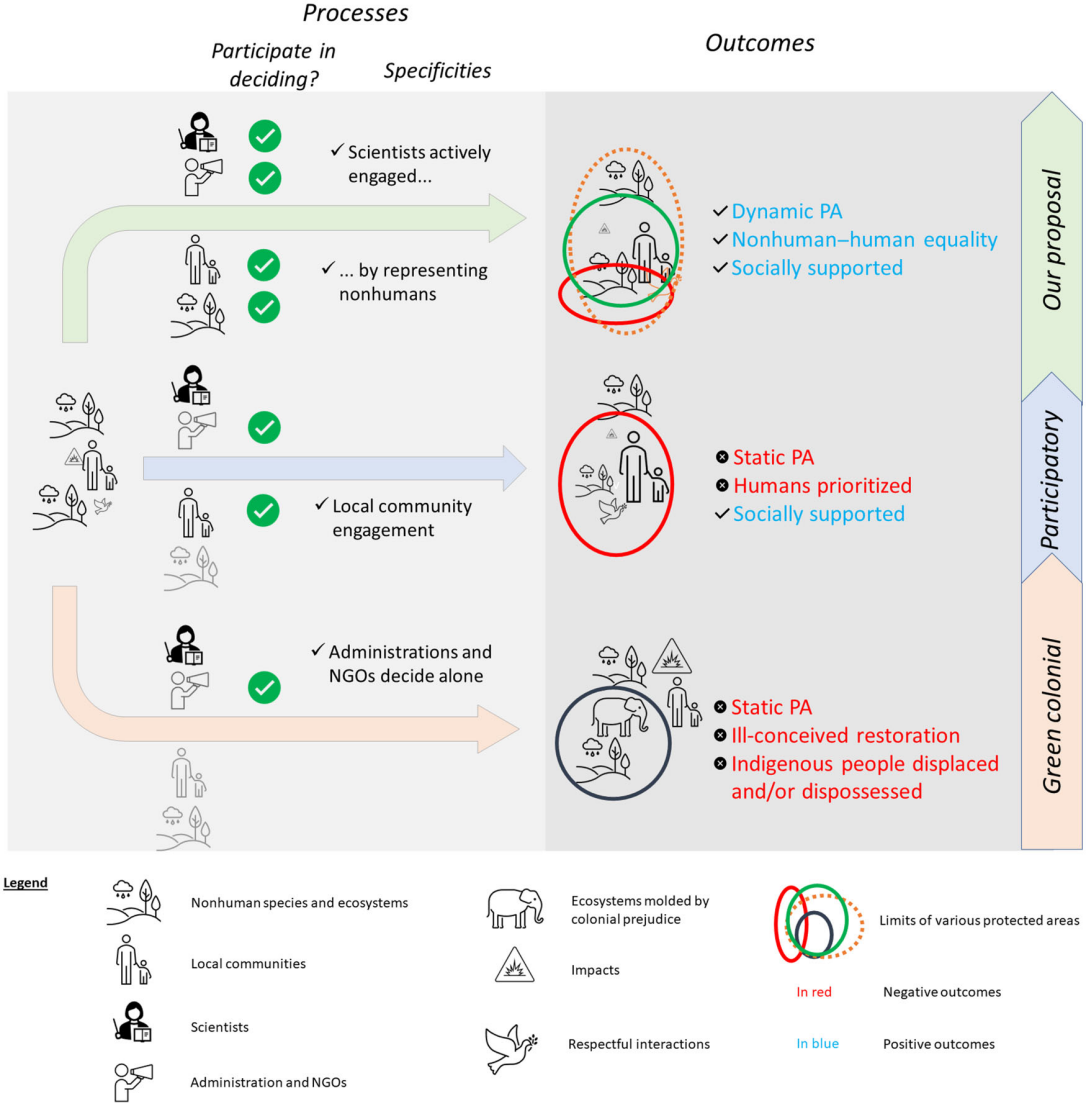
The process and expected outcomes of the proposed model (upper panel) for the definition and management of protected areas (PAs) compared with the participatory and green colonial models (NGO, nongovernmental organization; PA, protected areas).

Many environmental policies around the world still grant a major role in conservation to areas with geographically fixed frontiers (Zafra‐Calvo et al., 2019). The logic of fixing geographic frontiers for conservation purposes is, however, questionable on many counts (Brennan et al., 2022; Antonio de la Torre et al., 2022; Hilborn & Sinclair, 2021; Smith et al., 2023; Williams et al., 2023). Many so‐called protected areas have been denounced as paper parks, with geographic frontiers drawn on maps and no effective protection (e.g., Pieraccini et al., 2017). Besides, the literature on “green colonialism” (Blanc & Morrison, 2022; Grove, 1995) shows that colonialist prejudice has distorted the practice of delineating protected areas in many instances. An “artificial nature” has thereby been created by excluding Indigenous peoples from areas where they used to entertain durable interactions with their environment (Adams & McShane, 1997; West et al., 2006). This resulted in injustice and exacerbated conflicts (Figure 2, lower panel).

A different approach was used, in particular (but not only) in Europe, where some human activities are considered valuable contributions to biodiversity. Many national parks and nature reserves in Europe accordingly allow economic activities, including agricultural practices needed to preserve ecosystems, e.g., hay cutting, whose preservation is seen as a major target of biodiversity policies such as Natura 2000 (Evans, 2006). This alternative approach inevitably involves negotiations and trials of force between actors with diverging interests (Alphandéry & Fortier, 2001) when delineating protected areas and during their subsequent management. The corresponding increasing inclusion of a variety of stakeholders has taken the form of a participatory model (Figure 2, middle panel). The latter arguably helped (at least in some cases) reduce conflicts and foster respectful interactions between human beings and their environment (Salvatori et al., 2020).

Other examples of similar participatory models are the numerous initiatives involving Indigenous people in conservation research and actions around the world (Dawson et al., 2024; O'Bryan et al., 2021), through which a vast array of stakeholders can get involved in the definition of perimeters of protected areas and their subsequent management (Dawson et al., 2023; Sze et al., 2022; Tran et al., 2020). However, in applications of this model, socioeconomic, administrative, and political logics often take priority, and delimitations of protected areas therefore often end up being extremely unsatisfactory from a conservation point of view. Figure 1 illustrates this idea in the Maures Plain Nature Reserve. The Natura 2000 network of protected areas similarly provides countless examples. For instance, the Albanais Wetlands Network in France is composed of 42 insulated patches of wetlands scattered throughout dense urbanization and high‐yield farming. The latter is a constant source of major pollution in these supposedly protected areas, and there is no opportunity to dampen this pressure (Meinard et al., 2024). Because, by definition, OECMs are structured by objectives other than biodiversity protection, their delimitation can match biodiversity's requirements only fortuitously.

All in all, whether due to colonialist prejudice or administrative or socioeconomic logics, many protected areas have geographic frontiers that sideline the biological needs of target species and ecosystems and ignore the socioeconomic and physical logic underlying major environmental impacts. This is all the more true in the era of global anthropogenic activities (Maldonado‐Oré & Custodio, 2020; Semper‐Pascual et al., 2023; Titley et al., 2021).

Despite all these caveats, some protected areas have geographic frontiers that make good ecological sense. This is especially the case when these frontiers are based on important, impervious, geomorphologic features of the landscape that play key ecological roles. The Ngorongoro Conservation Area, whose frontiers run the perimeter of a vast crater (Homewood & Rodgers, 1991), is a case in point. More generally, the critique of geographic frontiers cannot meaningfully lead to the conclusion that conservation policies should evacuate all forms of geographic limits. Indeed, this would lead to the absurd conclusion that protections should be the same everywhere.

Critical discussions on geographic limits of protected areas should rather focus on their unavoidable permeability to protected entities and threats and on means to ensure their plasticity. The term *frontiers* hence again appears particularly relevant because geographic frontiers of protected areas are limits whose crossing in opposite directions is high stakes and whose definition is an ever‐evolving challenging horizon.

## SYNERGISTIC CROSSING OF FRONTIERS OF CONSERVATION

The term *frontiers* is also useful for identifying means to break out of the 3 assumptions in synergy. We devised an ambitious program to unfetter conservation initiatives to that end.

As argued above (“THE NATURAL–ANTHROPIC FRONTIER”), adequately addressing the stakes of crossings of the natural–anthropic frontier implies that nonhumans be actively involved in environmental decision‐making. Such an active involvement goes beyond simply using nonhumans as conservation actors, for example, by having sheep graze grasslands to conserve them. In such cases, nonhumans are taken as a means to achieve conservation objectives set by human decision makers. The active involvement of nonhumans rather requires generalizing, through new regulatory obligations, the dynamics that have gradually strengthened existing regulatory obligations to involve human stakeholders in environmental decision‐making processes since the 1990s (Blondet et al., 2017; Buchs et al., 2021). Such participatory obligations should be generalized to include actors (i.e., representatives) entrusted with the task to give a voice to nonhuman entities (i.e., represented ecosystems or populations of nonhuman species) (Edelblutte et al., 2023; Gray & Curry, 2020; Meinard et al., 2022) (Figure 2, upper panel).

This representative role is very different from the role of experts who use their knowledge to provide advice about how best to achieve decision makers’ objectives. Such experts do not participate in decision‐making about objectives, and decision makers can ignore their advices entirely. By contrast, participants in collective decision‐making can fight to make a difference. Representing nonhumans also involves—but goes beyond—recognizing natural entities’ right to be protected from destruction (Chapron et al., 2019; Clark et al., 2019; Eckstein et al., 2019; Gellers, 2021). By being represented in decision‐making, nonhumans would not only be protected from removal or destruction, but also would be in a position to foster actions they can benefit from, like human stakeholders do in participatory decision‐making. In concrete terms, this would take the form of efforts to implement ambitious protection, restoration, and reintroduction schemes that would compete with efforts by human stakeholders to monopolize land and resources.

In standard participatory environmental decision‐making, local environmental nongovernmental organizations (NGOs) sometimes already endorse representation of nonhumans. Similarly, in numerous conservation initiatives involving Indigenous peoples, their representatives contribute, among other things, to fostering recognition of nonhumans and their distinct interests (Fabiano et al., 2021; Popken et al., 2023; Thomas, 2015). However, not all conservation projects have such environmental NGOs nearby or involve representatives of Indigenous peoples. Besides, when they are present, such actors typically focus on a subset of concerned nonhumans. Therefore, they ignore many nonhumans that can benefit from or can be affected by planned actions. In any case, human groups are typically represented by actors that are either more numerous or more powerful or both than representatives of nonhumans.

The effective participation of nonhumans we call for requires a massive scaling up and generalization of such efforts at representing the vast diversity of concerned nonhumans. This involves on‐going challenges, as captured by the idea of a frontier as ever evolving. However, these problems are not specific to the representation of nonhumans, and standard participatory practice provides avenues to solve them.

A primary difficulty is that the recruitment of representatives can be time consuming and difficult (especially concerning poorly known species or ecosystems). However, as in standard participatory processes, prior studies of the natural context can be used to identify relevant knowledge holders who will provide feedback on issues neglected in existing studies, leading in turn to identifying additional relevant knowledge holders.

Once representatives are identified, ensuring that they represent nonhumans in a correct way is also challenging, especially given that, unlike human beings who are represented in participatory decision‐making, nonhuman entities can neither speak nor vote to express their assent or dissent. However, this concern should not be exaggerated. The fact that representation can never be perfect is established in standard participatory processes. It can be addressed, as in participatory practice, by opening the processes of representative recruitment and representation. In an open process, actors can point out problems (such as when a representative is biased or champions, for example, a colonialist worldview detrimental to local people), correct errors, or put forward better, alternative representatives.

Another challenge is how to give voice to antagonistic parties, such as prey species and their predators or other sets of represented nonhumans with antagonistic interests (e.g., native vs. non‐native species). Such situations already commonly happen in participatory decision‐making, where stakes are routinely organized as bundles, different representatives are associated with different bundles, and participants collectively strive to find compromises between antagonistic interests.

Another seeming obstacle is that it might look unrealistic to expect that all nonhuman stakeholders can be represented. Indeed, even in small‐scale projects, infinitely numerous species and ecosystems can be affected by or benefit from implemented actions. Here again, this worry exaggerates a problem that holds true for any decision or action, whatever the stakes and context. The relevant stakeholders to involve in decision‐making in a given situation can be identified on a pragmatic basis, through an open snowball process. This practice, which is standard in participatory decision‐making, can be generalized.

In this process of representation of nonhumans in decision‐making, conservation scientists have a key role to play. The involvement of many other knowledge holders, such as local experts in NGOs, consultancies, and environmental administrations and local people holding local or indigenous knowledge, should be encouraged, along with that of conservation scientists. However, because they have the relevant cutting‐edge knowledge and can contribute insights that are complementary to the equally legitimate and relevant insights from local knowledge, conservation scientists should at least take upon themselves a relatively large proportion of the workload. The currently vastly understaffed expert institutions involved in environmental decision processes would certainly welcome help from conservation scientists. Many conservation scientists already endorse similar tasks by acting as experts for NGOs or institutions (e.g., IPCC, IPBES, knowledge hubs) or by getting involved in conservation projects as Indigenous knowledge holders. Such mergers of identities should be generalized in upscaled and multiplied attempts at involving all the human and nonhuman stakeholders in collective decision‐making.

This recommendation is surely bound to collide with the fear that, by intervening in regulatory settings designed to represent nonhumans, scientists would lose their objectivity. Conservation scientists should consider the generalized representation of nonhumans in conservation decision‐making as an impetus and opportunity to cross the knowledge–activism frontier. Scientists should not be shy to cross this frontier because it can be a constant source for renewal of their critical reflections.

This new way of acting and deciding collectively with human and nonhuman beings paves the way for rethinking the geographic frontiers of protected areas, conceived as limits whose crossing in opposite directions is high stake and whose definition is an ever‐evolving challenging horizon. Indeed, the relevance of existing geographic limits is bound to be questioned in many instances if nonhumans are duly represented in decision‐making. Instead of taking geographic limits for granted, the approach we propose (Figure 2, upper panel) starts by convening, for the various conservation problems addressed, decision committees that include representatives of concerned humans and representatives of concerned nonhumans. Among the decisions that such committees should make, one should be to determine the temporal and spatial scope of the envisioned actions for tackling the problems addressed. The result will be multidimensional protected areas that are not only dynamically fine‐tuned to the specificities of the conservation issues they are designed to address but also dynamically responsive to the current rapid changes in environmental conditions.

Protected areas are already multilayered, with innumerable overlapping statuses (Deguignet et al., 2017) and differentiations of statuses within single protected areas (e.g., cores vs. buffers). Such differentiations are currently determined in collective decision processes in which nonhumans are poorly represented and conservation scientists act as advisors—if at all.

In line with the abovementioned need to group nonhumans in bundles with coherent stakes, the various projects to define dynamic protected areas devoted to addressing different conservation issues should be coordinated to identify synergies and merge projects when relevant in an ecosystem‐based approach (Radinger et al., 2023). Our proposed dynamic and flexible scheme would permit taking interactions and synergies into account in readjustments of perimeters. Because differentiations of protections would be facilitated when issues are too different, mergers of protections would similarly be facilitated. The fact that existing environmental policy tools and schemes often ignore interactions between different impacts and synergies in possible remedies will thereby be addressed.

The ensuing plasticity and dynamics of protection perimeters require drastic simplifications of the administrative processes through which perimeters of protected area are currently defined. More generally, for our recommendation to be implementable without involving an exponential increase in bureaucratic workload and associated inefficiencies, massive administrative, and regulatory simplifications are needed.

Moreover, in our scheme, because perimeters and types of protections will be multilayered and dynamic, possible conflicts with human stakeholders could multiply. Our proposal should not become a hindrance to progress in recognizing the rights and well‐being of Indigenous peoples and local communities (Reyes‐García et al., 2022). An unavoidable corollary is therefore that the generalization of participatory decision‐making involving nonhuman stakeholders should entail upscaling of the participation of human stakeholders (in line with recent recommendations of Newing et al. [2024]). This strengthened involvement of all stakeholders could ease social and economic conflicts, which can lead to secondary benefits for biodiversity.

## HOW TO GET THERE FROM HERE

Our proposal delineates a new future that is not only desirable but also operationally feasible. This operationalization involves 3 key steps: first, enactment of regulatory reforms that entrench the obligation to identify key conservation problems and that require steering committees to include human and nonhuman representatives; second, launch of the process that includes the involvement of conservation scientists, experts, stakeholders, and local people and subsequent definition of *dynamic protected areas*; third, maintenance of the dynamic scheme in which regular ecosystem monitoring and meetings are held that lead, when relevant, to new differentiations and mergers of protected areas and adjustments of perimeters.

The 3 steps are certainly difficult, time consuming, and costly to implement. However, they are not more so than current processes of creation and functioning of existing protected areas and other conservation policies. A reallocation of time, money, and energy to fuel the new scheme is liable to provide a substantive part of the needs.

The envisioned scheme could take various forms, depending on local political and socioeconomic contexts. Ideally, the starting point for identification of conservation problems should be done at a global scale so that global problems, such as climate change or biological invasions, can be addressed subsequent to identification of problems to be tackled at more regionalized scales. However, initiatives starting at regional scales may be more realistic and can be a first step in the right direction.

The active involvement of conservation scientists in the new representation processes we call for (second and third steps above) and their involvement in putting pressure on decision makers to implement the reforms we recommend (first step) require scientists to prioritize decision‐making at the expense of pure research. If the current quantitative production of scientific work is to be sustained despite the corresponding diversion of time and work, substantive additional funding and workforce among conservation scientists will be needed. Scientific institutions should also better value individual scientists’ involvement in decision‐making.

These and other regulatory and institutional changes will face obstacles associated with existing power relations (Devictor & Meinard, 2020; Jones, 2019). Devising strategies to overcome them is beyond the scope of this essay through which we sought to delineate a desirable and feasible future for conservation. Strategic questions about how to reach this future will only be relevant if it is endorsed as a desirable one by conservation scientists. Being mainly conceptual, our work at this stage also leaves untouched numerous intricate practical questions, in particular concerning specifics of the protocols to be elaborated to represent nonhumans in practice. Also beyond the scope of this essay is the question of the quantitative extent to which our proposal can improve conservation achievements. The answer to this question depends on concrete implementation specifics and involves intricate conceptual and technical choices concerning relevant metrics.
